# Transposition of the Teeth

**Published:** 1847-10

**Authors:** 


*Transposition of the Teeth.*—A young lady aged about eighteen, called upon us a few weeks since for the purpose of obtaining our professional services, and upon examining her teeth, we perceived that the right superior cuspidatus was situated between the central and lateral incisores of the same side, while the lateral was situated between it and the first bicuspis. The cuspidatus was well developed, and presented all the distinguishing characteristics which belong peculiarly to this class of teeth. The lateral incisor was of a medium size. In all other respects, the teeth were well arranged.

We have met with several similar examples of the transposition of teeth, and they have been noticed and described by several writers upon odontology. Miel presented two such examples to the Medical Society of Emulation. Delabarre has presented an engraving of one similar to the above. But how are such aberrations in the arrangement of the teeth caused? The generally received opinion upon the subject is, that they are the result of a transposition in the arrangement of the germs or pulps of these organs, and this would seem to be the only way by which such transposition could occur. But is it not possible, taking the case here described as an example, that the carneous body formed by the contraction of the sac of the permanent lateral incisor may have been developed on the side next the root of the temporary cuspidatus, and after having destroyed the intervening alveolar plate, that it then effected the destruction of the root of this tooth, instead of that of the temporary lateral incisor, and if so, is it not probable that it replaced the former, while the latter remained, as yet, undisturbed? Pursuing the supposition still further, might not the position of the cuspidatus have been so changed by the presence of the permanent lateral incisor, which had replaced the temporary cuspidatus, that the carneous body afterwards formed by the contraction of its sac, was made to act upon the root of the temporary lateral? and if so, is it not reasonable to presume, that the cuspidatus replaced the loss of the last named tooth?

That the transposition of permanent teeth is thus caused, we are not prepared to maintain. We throw out the suggestion merely for the purpose of eliciting inquiry upon the subject, and, in this connection, we would ask, if it was the result of a transposition of the germs of the teeth, would not the same aberration be as likely to occur in the arrangement of the temporary as in the permanent teeth?—***Balt. Ed.***

